# CSB-PGBD3 Mutations Cause Premature Ovarian Failure

**DOI:** 10.1371/journal.pgen.1005419

**Published:** 2015-07-28

**Authors:** Yingying Qin, Ting Guo, Guangyu Li, Tie-Shan Tang, Shidou Zhao, Xue Jiao, Juanjuan Gong, Fei Gao, Caixia Guo, Joe Leigh Simpson, Zi-Jiang Chen

**Affiliations:** 1 Center for Reproductive Medicine, Shandong Provincial Hospital Affiliated to Shandong University, National Research Center for Assisted Reproductive Technology and Reproductive Genetics, The Key Laboratory for Reproductive Endocrinology of Ministry of Education, Jinan, China; 2 State Key Laboratory of Biomembrane and Membrane Biotechnology, Institute of Zoology, Chinese Academy of Sciences, Beijing, China; 3 State Key Laboratory of Reproductive Biology, Institute of Zoology, Chinese Academy of Sciences, Beijing, China; 4 Key Laboratory of Genomic and Precision Medicine, Beijing Institute of Genomics, Chinese Academy of Sciences, Beijing, China; 5 Research and Global Programs March of Dimes Foundation, White Plains, New York, United States of America; 6 Renji Hospital, Shanghai Jiao Tong University School of Medicine, Shanghai, China; Stanford University School of Medicine, UNITED STATES

## Abstract

Premature ovarian failure (POF) is a rare, heterogeneous disorder characterized by cessation of menstruation occurring before the age of 40 years. Genetic etiology is responsible for perhaps 25% of cases, but most cases are sporadic and unexplained. In this study, through whole exome sequencing in a non-consanguineous family having four affected members with POF and Sanger sequencing in 432 sporadic cases, we identified three novel mutations in the fusion gene *CSB-PGBD3*. Subsequently functional studies suggest that mutated CSB-PGBD3 fusion protein was impaired in response to DNA damage, as indicated by delayed or absent recruitment to damaged sites. Our data provide the first evidence that mutations in the CSB-PGBD3 fusion protein can cause human disease, even in the presence of functional CSB, thus potentially explaining conservation of the fusion protein for 43 My since marmoset. The localization of the CSB-PGBD3 fusion protein to UVA-induced nuclear DNA repair foci further suggests that the CSB-PGBD3 fusion protein, like many other proteins that can cause POF, modulates or participates in DNA repair.

## Author Summary

Through whole exome sequencing in a non-consanguineous family having four affected members with POF and Sanger sequencing in 432 sporadic cases, we identified three novel mutations in *CSB-PGBD3*. Our functional studies implicate *CSB-PGBD3*, a gene which has previously shown association with DNA repair and Cockayne syndrome has a potential role in maintenance of ovarian function. This study also provides evidence for a pivotal role of DNA repair in POF.

## Introduction

Premature ovarian failure (POF), also known as premature ovarian insufficiency (POI), typically is defined by elevated serum FSH levels prior to the age of 40 years [1]. Approximately 1% of women are affected. The disorder is heterogeneous, causation including chromosomal abnormalities and single gene mutations, as well as autoimmune, metabolic, infectious and iatrogenic factors [1]. Evidence for genetic factors has been provided by population and candidate gene studies. Approximately 10 − 15% of cases have an affected first or second degree relative [2], although proven genes with functional confirmation exist only for *FMR1*, *NR5A1*, *BMP15*, *NOBOX*, *FIGLA*, *PGRMC1* and *GDF9* [3–10]. Genome wide association studies (GWAS) have revealed multiple loci potentially associated with POF in Chinese, Korean, and Dutch [11–13]. However, in each it was difficult to implicate specific novel genes, and the positive findings were not always replicated. Recently, some causative perturbation has been found in POF associated with somatic anomalies, such as Perrault syndrome and blepharophimosis-epicanthus syndrome type 1 (BPES1), using whole genome or exome sequencing [14–16]. However, low prevalence and impaired fecundity result in limited pedigrees of POF without associated somatic anomalies (non-syndromic), and whole exome sequencing has not yet been conducted in non-syndromic POF kindreds having more than one affected member until the recent report by Wang, in which compound heterozygous mutation in the HFM1 gene were identified [17].

Here, we reported our results of whole exome sequencing in a Chinese non-consanguineous POF kindred and Sanger sequencing in 432 sporadic POF patients. We identified one heterozygous *PGBD3* mutation in the kindred, and two *CSB-PGBD3* fusion gene mutations among the 432 sporadic patients. All three mutants of *CSB-PGBD3* were impaired in DNA damage repair, as indicated by delayed or absent recruitment to sites of cellular damaged. This could be caused by dysfunctional interaction with RNA polymerase II (RNAPol II). However, other mechanisms could exist.

## Results

### Patients with POF in the non-consanguineous family

**Fig 1:**
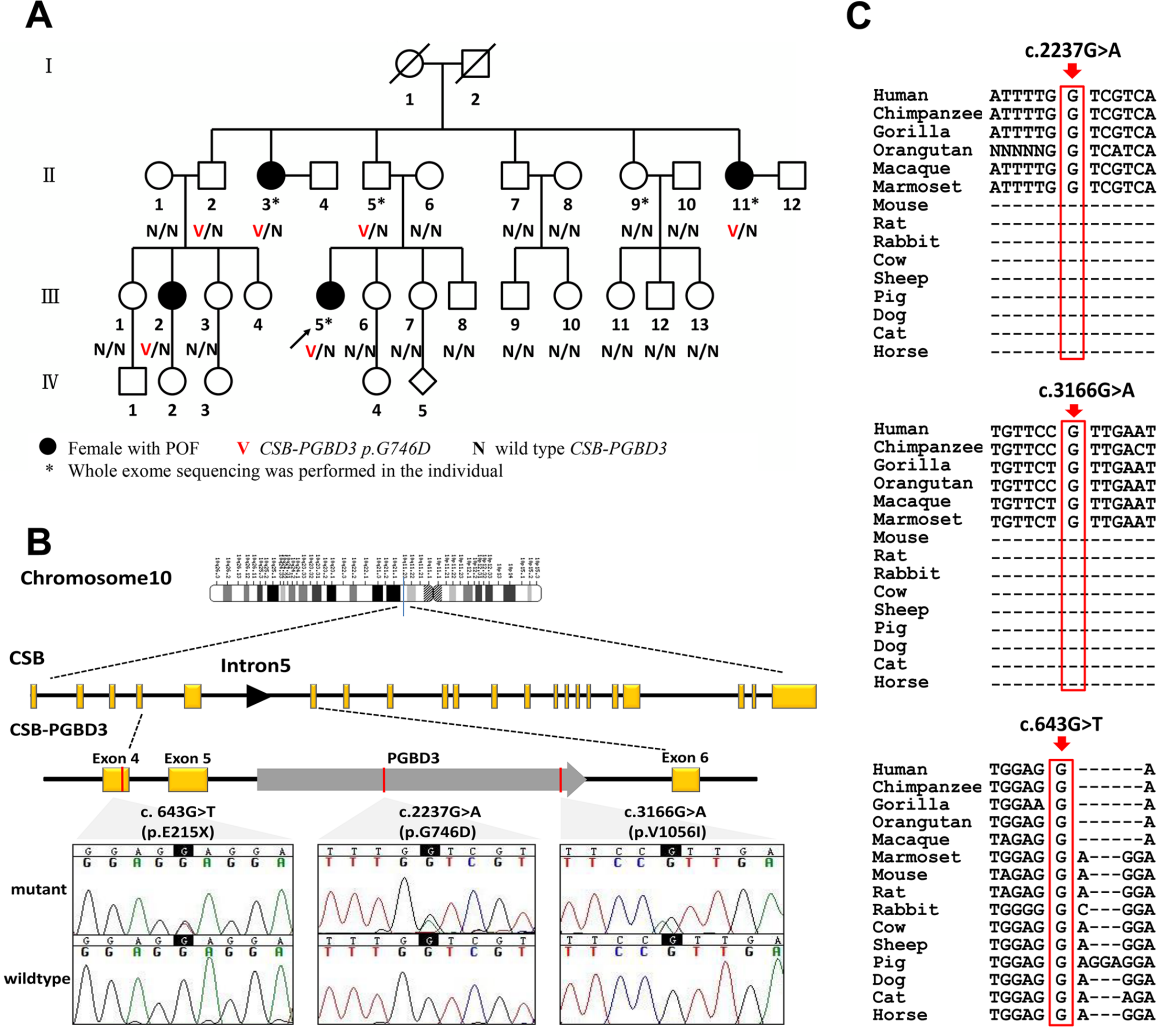
CSB-PGBD3 mutations identified in the index family with POF and sporadic cases. (A) Pedigree of the index family, ascertained through Ⅲ 5. Whole exome sequencing was performed in POF patientsⅡ3, Ⅱ11 and Ⅲ5; a genetically obligate male carrier (Ⅱ5); and one normal family member Ⅱ9. Sanger sequencing of CSB-PGBD3 was performed in other family members when DNA was available. (B) Genomic structure of CSB-PGBD3 and chromatograms of three mutations identified in present study. PGBD3 was inserted into intron 5 of CSB to make the fusion gene CSB-PGBD3. CSB exons are in yellow, PGBD3 exon in gray, and the three mutations indicated in red, and the corresponding chromatograms are shown. (C) Alignment of the coding strand of CSB-PGBD3 in 15 eutherian mammals from Ensembl database shows conservation of nucleotides 2237 and 3166 in primates and 643 in mammals.

The index case (Fig 1A, Ⅲ5) in the family was a 28-year-old woman of Han Chinese descent who presented with secondary amenorrhea at 23 years of age, with serum follicle stimulating hormone (FSH) concentrations exceeding 40 IU/L on two occasions. In her family there were 3 other females with POF. Ⅱ3 andⅡ11, currently in their 60s or 50s, were childless and presented with secondary amenorrhea at the age of 18 years and 27 years, respectively. Ⅲ2 experienced oligomenorrhea from the age of menarche at age 14 years, gave birth at age 30 years to a normal infant after attempting, unsuccessfully, for the previous 6 years. She developed amenorrhea at the age of 37 years. Chromosomal abnormalities, *FMR1* premutation, previous ovarian surgery, or exposure to chemotherapy or radiotherapy were not present in any family member.

### Whole exome sequencing detects a heterozygous missense mutation in *PGBD3*

Whole exome sequencing was performed in the three known affected casesⅡ3, Ⅱ11 and Ⅲ5; one genetically obligate male carrier (Ⅱ5) should a mutation be segregating in autosomal dominant fashion; and one normal family member Ⅱ9. More than 20,000 single-nucleotide variants (SNVs) were identified in each subject. After being “blasted” with public databases, verified by Sanger sequencing, and sequencing the remaining family members (Ⅱ1, Ⅱ2, Ⅱ6, Ⅱ7, Ⅱ8, Ⅱ10, Ⅲ1, Ⅲ2, Ⅲ3, Ⅲ6, Ⅲ7, Ⅲ8, Ⅲ9, Ⅲ10, Ⅲ11, Ⅲ12, Ⅲ13), only the heterozygous variant (ENST00000515869: c.2237G>A, p.G746D) in PiggyBac transposable element derived 3 (*PGBD3*) was exclusively carried by those affected individuals (Ⅱ3, Ⅱ11, Ⅲ2 and Ⅲ5), and the obligate carrier (Ⅱ5), and absent in normal family members. Ⅲ2 and Ⅲ5 inherited the variant from their fathersⅡ2 and Ⅱ5, respectively, supporting autosomal dominant inheritance (Table 1 and Fig 1).

**Table 1:** Clinical features of index family and 2 sporadic POF patients with mutations in *CSB-PGBD3*.

Patient No.	*CSB-PGBD3* Mutation		Genotype	Age (yr)		Menstrual History		Age at Diagnosis (yr)	Prior Hormonal Treatment	Childbearing
	sequence variation	Amino acid variation				Age at Menarche (yr)	Age of Amenorrhea (yr)			
**Index Family**										
**Ⅱ3**	c.2237G>A	p.G746D	AG	66		14	18	29	No	None
**Ⅱ11**	c.2237G>A	p.G746D	AG	58		14	27	30	No	None
**Ⅲ2**	c.2237G>A	p.G746D	AG	36		14	37	37	Yes*	Conceived naturally and gave birth to a normal girl after 6 years of marriage and attempting to conceive
**Ⅲ5**	c.2237G>A	p.G746D	AG	28		13	23	28	Yes *	None
**Sporadic POF**										
**Case 1**	c.643G>T	p.E215X	GT	25		14	24	25	Yes *	None
**Case 2**	c.3166G>A	p.V1056I	AG	27		15	25	26	Yes *	None

*1–2 mg daily dose of oral estradiol valerate tablet for 21 days plus oral micronized progesterone (200 mg/d) for 12 days each month.

### Mutational screening in sporadic POF patients

To validate exome data and detect independent mutations, we further performed Sanger sequencing of *PGBD3* in 432 sporadic POF patients and 400 matched control females. Clinical features of the two cohorts were shown in Table 2. The *PGBD3* transposon integrated into intron 5 of the Cockayne syndrome Group B gene (*CSB*, also known as *ERCC6*, excision repair cross-complementing rodent repair deficiency, complementation group 6) about 43 Mya in marmoset. This resulted in abundant CSB-PGBD3 fusion protein arising by alternative splicing of *CSB* exons 1–5 to the *PGBD3* transposase which was conserved in the subsequent primate radiation [18]. Given that, we sequenced exons 1–5 of *CSB* in the two cohorts as well. As a result, we found one additional novel heterozygous missense mutation (ENST00000515869: c.3166G>A, p.V1056I) in *PGBD3* and one heterozygous nonsense mutation (ENST00000515869: c.643G>T, p.E215X) in exon 4 of *CSB* in POF patients; the latter resulted in a truncated protein without PGBD3. Neither perturbation was found among controls (S1 Table). The women having these two novel variants experienced normal puberty and established regular menses with menarche at 14 and 15 years old, respectively. They presented with secondary amenorrhea at the age of 24 and 25 years respectively, and neither of them had achieved pregnancy. They were of normal height and intellectually normal. Karyotype and FMR1 premutation analysis were normal. Somatic anomalies were not present in any family members. Clinical characteristics of mutation carriers were shown in Table 1.

**Table 2:** Clinical features of sporadic patients with POF and matched controls.

	Sporadic POF	Control
**Number of Cases**	432	400
**Age (yrs)**	32.07 ± 4.36	30.35 ± 4.50
**Age at menarche (yrs)**	14.5±1.8	14.8±2.7
**Age at amenorrhea**	25.1±5.6	NA
**Basal FSH (IU/L)**	77.0±27.4	6.76 ± 1.80

NA = not available

### Expression of CSB-PGBD3 in the primate oocyte

**Fig 2:**
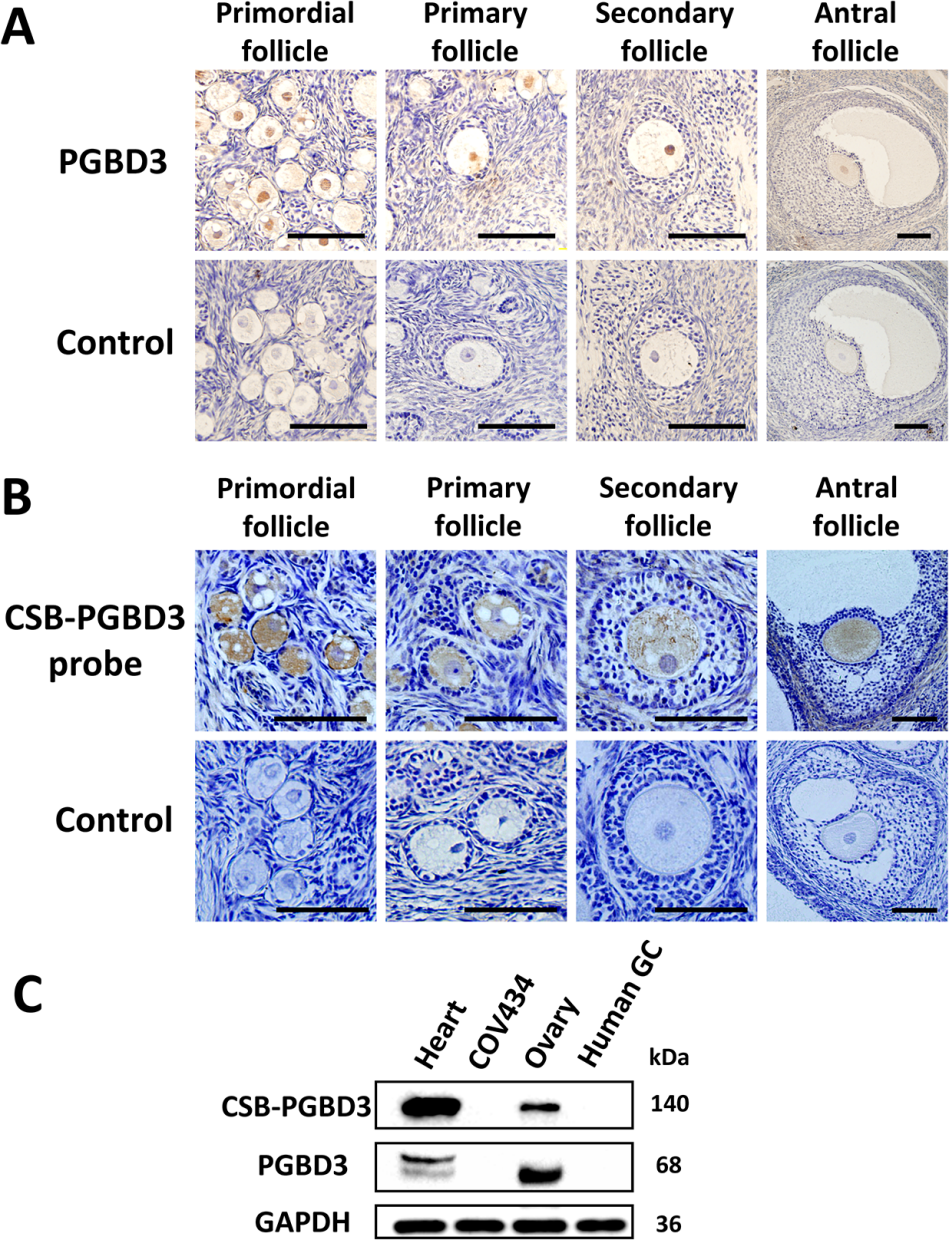
The cellular localization of PGBD3 and CSB-PGBD3. (A) Immunohistochemistry using anti-PGBD3 antibody (Abnova PAB21786) and (B) in situ hybridization using probe targeting at CSB-PGBD3 mRNA were performed in rhesus monkey ovary, which showed that CSB-PGBD3 was expressed exclusively in nuclei of oocytes from primordial to antral follicles. (C) Western blot performed with the use of anti-PGBD3 antibody (Abnova PAB21786) in human heart tissue, COV434 cells, adult ovary tissue and granulosa cells showed that PGBD3 and CSB-PGBD3 fusion protein were expressed in human ovary but not in granulosa cells. (Scale bars: 100 um.)

Through immunohistochemistry using anti-PGBD3 antibody (Abnova PAB21786) and through in situ hybridization using probe targeting at *CSB-PGBD3* mRNA on sections of formalin-fixed and paraffin-embedded ovarian tissue of rhesus monkey (9 years old), we observed that CSB-PGBD3 was exclusively expressed in nuclei of oocytes from primordial, primary, secondary to antral follicles (Fig 2A and 2B). Western blot with anti-PGBD3 antibody (Abnova PAB21786) in human ovarian tissue, granulosa cells, heart tissues and COV434 cells had confirmed the protein’s localization (Fig 2C), indicating that the CSB-PGBD3 fusion protein might play a role in oogenesis or maintenance of genomic stability of oocytes.

### Delayed recruitment of CSB-PGBD3 mutants at sites of DNA damage

**Fig 3:**
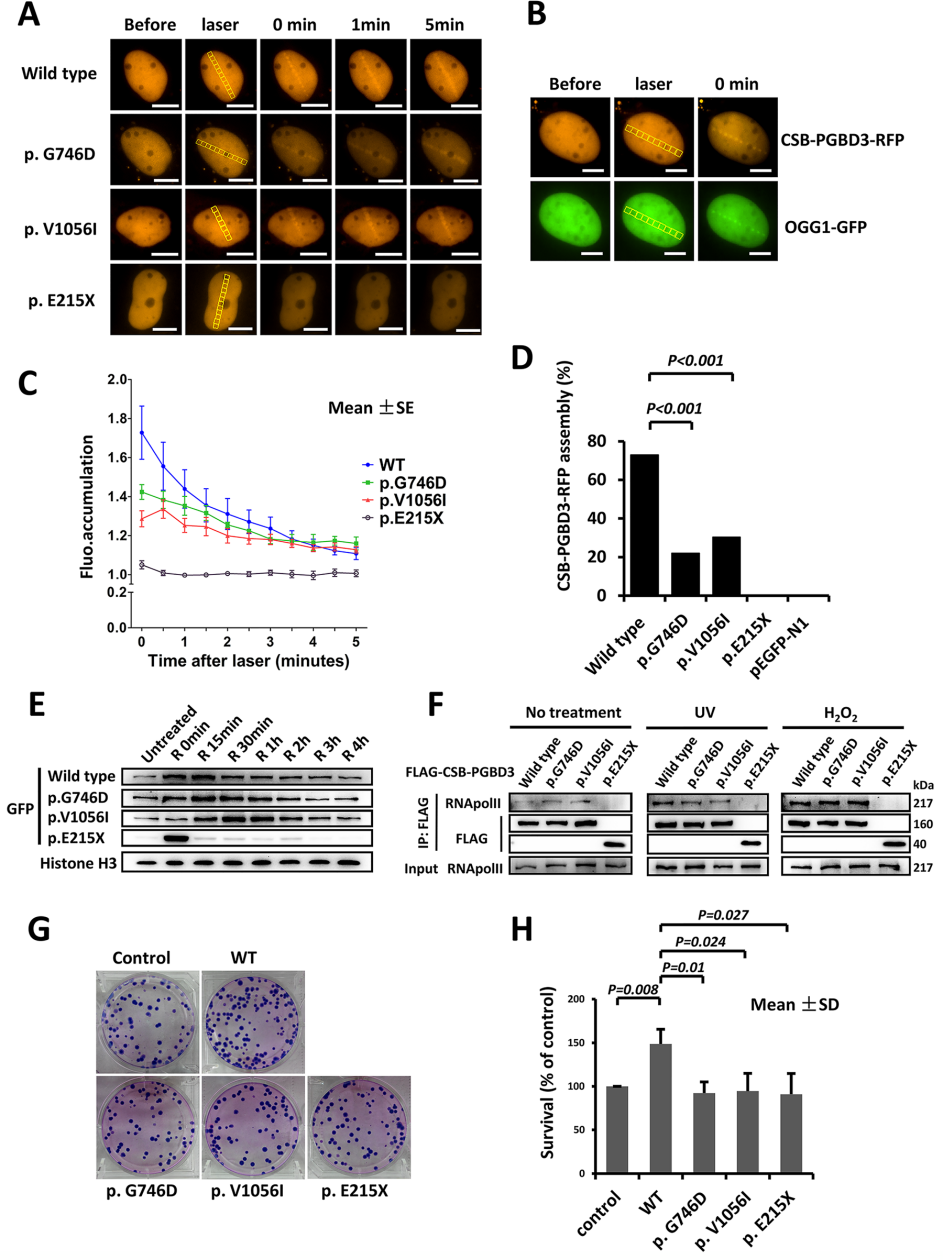
Delayed recruitment of CSB-PGBD3 mutants at DNA lesion. (A) DNA damage was induced by laser micro-irradiation in the nuclei of U2OS cells expressing wild type and mutant CSB-PGBD3-RFP. (B) OGG1-GFP was recruited to the DNA damage site along with CSB-PGBD3-RFP. (C) Fluorescence accumulation curve showed the recruitment of mutant p.G746D and p.V1056I to damaged sites were weaker compared with wild type, and mutant p.E215X could not accumulate at the damaged site completely. (D) Percentage of reactive cells after laser micro-irradiation was less for mutants compare with wild type (*p<0.001). (E) Western blot for wild type and mutant CSB-PGBD3-eGFP protein attached to chromatin in HeLa cells after H_2_O_2_ treatment. Mutant p.G746D and p.V1056I bound onto chromatin with a delayed time course compared with wild type. The quick recruitment and separation of p.E215X was elusive. (F) Immunoprecipitation experiments showed the truncated protein p.E215X failed to interact with RNApol II with or without treatment. (G) and (H) showed the clonogenic survival of CSB-PGBD3 tranfected cells suffering UV irradiation. The percent survival relative to the control was significantly higher for wild type compared with the mutants. (Scale bars: 5um)

U2OS and HeLa cells expressing CSB-PGBD3-RFP or CSB-PGBD3-eGFP (including wild type and three mutants) were exposed to laser micro-irradiation or oxidative damage. Similar to OGG1 (8-Oxoguanine DNA Glycosylase1, responsible for the excision of 8-oxoguanine in response to oxidative damage), which localizes to the laser damaged sites rapidly, wild type CSB-PGBD3-RFP protein was recruited to the damaged sites immediately after laser irradiation, and gradually disappearing around 5 min (Fig 3A and 3B). Similar response was observed after oxidative damage, in which CSB-PGBD3-eGFP bound onto chromatin immediately after H_2_O_2_ treatment and began to separate after 15 min (Fig 3E). Compared to the wild type, the response of mutants p.G746D and p.V1056I to DNA damage was much weaker (Fig 3C), and the percentage of cells containing CSB-PGBD3-RFP that were recruited to laser damaged sites was significantly lower (22.03% vs. 72.94%, p<0.001 for p.G746D; 30.30% vs. 72.94%, p<0.001 for p.V1056I; respectively) (Fig 3D). For the truncated protein p.E215X, no accumulation was observed at the laser damaged sites (Fig 3A, 3C and 3D). However, p.E215X bound onto chromatin immediately after H_2_O_2_ treatment but separated from it unexpectedly rapidly (Fig 3E). Protein p.E215X manifested no aggregation in 15 min after H_2_O_2_ treatment, which was the peak point for the recruitment of wild type, suggesting that p.E215X may not participate in the DNA damage repair.

It is known that CSB is among the first protein to bind stalled RNA polymerase II (RNApol II) at the sites of DNA damage, and is required to recruit other transcription-coupled DNA repair (TCR) factors [19]. CSB-PGBD3 fusion protein has been previously shown to interact with RNApol II as well. To verify previous findings and explore defects of identified three mutants, we performed immunoprecipitation (IP) between FLAG-CSB-PGBD3 (including wild type and mutants) and RNApol II. No significantly different interaction with RNApol II was observed among p.G746D, p.V1056I and wild type either after UV or H_2_O_2_ damage. However, the truncated protein p.E215X failed to associate with RNApol II either after UV or H_2_O_2_ damage (Fig 3F). Our clonogenic survival assay further showed that the clonogenic survival percent of wild-type CSB-PGBD3-expressing cells was significantly higher than that of mutants-expressing cells (Fig 3G and 3H), indicating a crucial role of functional CSB-PGBD3 played in maintaining cell survival after DNA damage. Taken together, our results suggest that CSB-PGBD3 could play a role in DNA damage repair, and that the mutations we identified impair this normal function of CSB-PGBD.

## Discussion

Whole exome sequencing in a kindred having multiple family members with non-syndromic POF identified the p.G746D mutation in *CSB-PGBD3*, inherited in autosomal dominant fashion. Among 432 sporadic POF cases, we discovered one additional novel missense mutation (p.V1056I) and one nonsense mutation (p.E215X) in *CSB-PGBD3*. Thus, this gene complex needs to be intact for normal ovarian development and maintenance.

CSB is an important player in TCR pathway. If perturbed, this results in Cockayne Syndrome (CS), an autosomal recessive disorder characterized by progressive growth failure, microcephaly, and mental retardation. In CS a dysfunctional DNA repair mechanism exists, nearly 70% of CS caused by perturbation in the *CSB* gene [20]. PGBD3 and CSB-PGBD3 fusion protein arose in the common ancestor of humans and marmosets about 43 million years ago and has been highly conserved ever since [18]. CSB-PGBD3 has been proved to participate in TCR of DNA damage [21]. However, there is no clear mechanism elucidating the role of PGBD3 plays in CS, nor has a mutation in *PGBD3* been identified in CS.

Curiously, complete absence of detectable CSB protein does not invariably cause CS: a nonsense mutation at codon 77 in CSB exon 2 causes only mild UV-sensitive syndrome (UVSS) with no syndromic or developmental components [20], whereas complete absence of CSB resulting from deletion of 5' UTR sequences including exon 1 causes classical early onset CS [22]. Indeed, it may be functionally significant that the novel nonsense mutation p.E215X in our sporadic POF patient, who had no CS, UVSS or other somatic anomalies, has never been found to cause CS [20].

Our finding- perturbations and functional alterations in POF cases- replies that CSB-PGBD3 play a role in ovarian development or maintenance. In the functional study, in response to the output of laser power that induced CSB-PGBD3-RFP to the damage site, OGG1-GFP was recruited to the damage site, indicating that CSB-PGBD3 is recruited to the UVA laser-induced oxidative damage sites, and the presence of functional CSB does not displace the fusion protein from the foci. There are several possible roles played by the proteins localized to DNA damage loci, including direct participants, indirect facilitators, or bystanders. Our study and others indicate that CSB-PGBD3 binds with RNApol II after DNA damage treatments. Combined with the fact that overexpression of CSB-PGBD3 promoted cellular resistance to UV irradiation, it is likely that CSB-PGBD3 is involved in DNA repair.

CSB-PGBD3 is known to interact with RNApol II through the N-terminal CSB domain [23]. In our study, the truncated protein E215X (nonsense mutation in CSB exon 4) manifested no binding with RNApol II and no accumulation in the laser damaged site, indicating that CSB-PGBD3 also requires exon 5 to interact with RNApol II. Mutant p.G746D, p.V1056I, and p.E215X did not change the nuclear localization as did wild type, but their response to DNA damage caused by laser/UV/H_2_O_2_ was delayed or absent. These results, in aggregate, suggest that CSB-PGBD3 fusion protein may be involved in DNA damage repair and three mutants could be loss of function partly or totally during the process. Therefore, haploinsufficiency of functional CSB-PGBD3 fusion protein might explain POF. However, at least 18 distinct homozygous or compound heterozygous mutations in CSB exons 1–5 are responsible for CS, and there is no observation of POF in any of the families of affected CS individuals, in which one would expect heterozygous women [20, 24]. More likely, the two novel sporadic mutations we observed (E215X and V1056I) acted in a dominant negative fashion.

In the present study, we demonstrated that CSB-PGBD3 fusion protein was expressed in oocyte, but not in granulosa cells or theca-interstitial cells. Of relevance, DNA damage is one of the most common insults that challenge oocytes [25]. Ability to repair DNA damage is essential to maintain the supply of oocytes necessary for reproduction. Many DNA repair genes are found to be expressed in oogenesis, such as *BRCA1* [26], *HELQ* [27] and *EXO1* [28]. If defects occur along the pathway of DNA repair, DNA damage could accumulate; oocytes may also be particularly vulnerable to ubiquitous external damage. Subsequently, the number of damaged follicles may increase with aging, leading to a greater rate of follicle loss through atresia, finally causing POF. Our results suggest that oocytes carrying mutant p.G746D, p.V1056I, and p.E215X either alone or together are unable to repair DNA damage efficiently. This provides direct genetic evidence that perturbation of genes involved in DNA damage repair could lead to non-syndromic POF. Our finding is consistent with recent reports that perturbations of MCM8 and MCM9 are responsible for POF, acting through compromised DNA repair [29,30].

In summary, we found perturbations of CSB-PGBD3 in familial and sporadic patients with POF. Functional studies involving CSB-PGBD3 can provide explanations relevant to ovarian maintenance.

## Materials and Methods

### Subjects and clinical data

A non-syndromic, non-consanguineous Chinese family having four POF members (Fig 1A). In addition, 432 independent Chinese sporadic POF patients and 400 matched Chinese control females were recruited between Jan 1, 2009 and Sep 1, 2012 from the Center for Reproductive Medicine, Provincial Hospital Affiliated to Shandong University. Inclusion criteria for all POF cases consisted of cessation of menstrual cycles before age of 40 years, with serum follicle stimulating hormone (FSH) concentrations on two occasions exceeding 40 IU/L. Women with known chromosomal abnormalities, FMR1 premutation, previous ovarian surgery, or exposed to chemotherapy or radiotherapy were excluded. Written informed consent was obtained from all participants. This study was approved by the Institutional Review Board of Reproductive Medicine of Shandong University ([2012] IRB No.16). Clinical characteristics of sporadic patients with POF and controls are shown in Table 2.

### Tissues and cells

The human fetal heart tissue was obtained from a fetus undergoing pregnancy termination at 28 weeks; adult ovary tissue was obtained from a woman (39 years old) undergoing oophorectomy because of ovarian serous cystadenocarcinoma; human granulosa cells were obtained from follicular fluid of a patient with tubal factor necessitating in vitro fertilization. All the human tissues and cells were obtained from Department of Gynaecology and Obstetrics and Center for Reproductive Medicine, Provincial Hospital Affiliated to Shandong University, with ethical approval under Codes of Practice of the China Human Tissue and study approval by the Institutional Review Board of Reproductive Medicine of Shandong University ([2013] IRB No.4).

Human cell lines: HeLa (Human cervix carcinoma cell line), U2OS (Human osteosarcoma cell line), HEK293FT (human embryonic kidney cell line) and COV434 (Human ovarian granulosa tumour cell line) were cultured in DMEM/High Glucose (Thermo) supplemented with 10% fetal bovine serum at 37°C with 5% CO_2_.

### Exome sequencing and data analysis

Genomic DNA was extracted from peripheral blood leukocytes using DNeasy Blood & Tissue Kit (Qiagen), exome sequences were captured with SureSelect Target Enrichment System for Illumina Paired-End Sequencing Library (Agilent Technologies), and DNA sequencing was performed on the Illumina platform (Illumina HiSeq). Reads were mapped to the hg19 reference genome with Burrows-Wheeler Alignment tool (BWA), and variants were called and annotated using GATK, ANNOVAR, and custom pipelines. Protein-coding variants were checked against established databases (1000 Genomes Project and dbSNP, version 134). All unreported variants following an autosomal dominant inheritance model were confirmed in the 5 subjects by Sanger sequencing, and the candidate variants were further validated in other individuals of the family by Sanger sequencing.

### Western blot

Tissues grinded and cells harvested by scraping were resuspended and lysed in 200 μl Radio Immunoprecipitation Assay (RIPA) buffer with 1mM Phenylmethanesulfonyl fluoride (PMSF) (Beyotime), and centrifuged in 12000 rpm at 4°C for 10 min. The supernatants were collected and protein concentrations were measured by the BCA method. Equivalent amounts of protein were separated by 8% SDS-PAGE gel and electro-transferred to PVDF membranes. The PVDF membranes were blocked with 5% nonfat milk in TBST for 2 h at room temperature and then incubated with primary antibodies overnight at 4°C. The primary antibodies used were as follows: PGBD3 (Abnova PAB21786, 1:500), RNApol II (Santa Cruz Biotechnology sc-9001, 1:1000), GFP (Proteintech 66002-1-lg, 1:5000), FLAG (Cell Signaling Technology 2368, 1:1000), β-Actin (Sigma-Aldrich SAB1305546, 1:5000), GAPDH (Sigma-Aldrich SAB2103104, 1:10000) and H3 (Merck Millipore 05–499, 1:100000). Membranes were washed with TBST, incubated with HRP-conjugated anti-rabbit or anti-mouse secondary antibody for 1 h at room temperature, and subjected to chemiluminescent detection with ChemiDoc MP System (Bio-Rad).

### Immunohistochemistry

An ovary from a rhesus monkey (9 years old) was fixed in 10% neutral formalin, embedded in paraffin and sectioned at 5 um thickness. Immunohistochemistry was performed using the Goat ABC Staining System (Santa Cruz Biotechnology). Briefly, the sections were deparaffinized completely and then immersed in citric acid buffer (10 mmol/L of sodium citrate, 10 mmol/L of citric acid) and boiled in water bath at 92–98°C for 15 min to expose the antigens. Sections were cooled to room temperature and sequentially permeabilized with 0.3% Triton X-100, incubated with 3% H_2_O_2_ at 37°C for 15 min to quench endogenous peroxidase, blocked with normal goat serum for 2h, incubated with anti-PGBD3 antibody (Abnova PAB21786, 1:20) at 4°C overnight, incubated with biotinylated secondary antibody at 37°C for 1 h and finally treated with the Streptavidin-HRP for 1 h. Intervening PBS washes were performed after incubation when necessary. Sections were then stained with diaminobenzidine (DAB) and hematoxylin, dehydrated and mounted with coverslips. Primary antibodies were replaced by PBS in negative controls. Signals were recorded with an Olympus IX51 digital camera system (Olympus).

### In situ hybridization

CSB-PGBD3 probe 5’- GAGGCATCTTGGGACTTAACCGCTGCTTA -3’ was labeled with DIG by using the DIG Oligonucleotide Tailing Kit (Roche). Sections of formalin-fixed and paraffin embedded ovary tissue of rhesus monkey (9 years old) was deparaffinized and washed with 0.1M Phosphate buffered saline treated with 0.1% diethylpyrocarbonate (DEPC-PBS). Then the sections were treated with 0.3% Triton X-100 for 15 min, incubated with 10 ug/ml Proteinase K (diluted in 50 mM Tris with 2 mM Calcium Chloride) for 15 min, fixed with 4% p-formaldehyde/0.1M PBS for 15 min at room temperature, and incubated with 0.25% acetic anhydride for 10 min. Intervening DEPC-PBS washes were performed after incubation when necessary. Then, the sections were incubated with hybridization buffer at 37°C for 2 h, washed with 2X SSC for 5 min, and incubated with 200 ng/ml of DIG-labeled CSB-PGBD3 probe at 37°C overnight. After hybridization, sections were sequentially washed in SSC and washing buffer [100 mM Tris-HCl (pH7.5), 150 mM NaCl], blocked with 2% normal goat serum for 30 min, and incubated with the diluted anti-DIG antibody (1:200) for 4 h at room temperature. Then sections were stained with BCIP/NBT and methylene green, and mounted with coverslips. The oligo probe of comparable length but scrambled sequence was used as a negative control: 5’-TGTAGTGCGGAATCGG CCTTATGCAACCT-3’. Signals were recorded with an Olympus IX51 digital camera system (Olympus).

### Laser micro-irradiation and imaging

U2OS cells were cultured and transiently transfected with wild type and the 3 mutant CSB-PGBD3-RFP plasmids together with or without OGG1-GFP plasmid using Lipo2000 (Invitrogen). DNA damage was induced in the nuclei of cultured cells by micro-irradiation with a pulsed nitrogen laser (Photonics Instruments; 365 nm, 10 Hz pulse) [31]. Briefly, cells were seeded onto 35-mm glass bottom dishes (MatTek) overnight before being visualized with a Nikon Eclipse Ti-E inverted microscope equipped with a computer-controlled MicroPoint laser Ablation System (Photonics Instruments) for time-lapse imaging. The output of the laser power was set at 70–90% of the maximum as indicated by the manufacturer. During micro-irradiation and imaging, cells were maintained at 37°C. The growth medium was replaced by CO_2_-independent medium (Invitrogen) before analysis. The mean fluorescence intensity of CSB-PGBD3-RFP foci in the laser induced damage sites and that of the other nuclear area were determined after subtraction of the extranuclear background signal. The ratio of the foci fluorescence intensity over the other nuclear area fluorescence intensity was used to indicate the quantification of the recruitment. The average and SE of a total of at least 10 cells are depicted in the graph.

### Clonogenic survival

HeLa cells were cultured and CSB-PGBD3 fusion protein silenced with siRNA (S5 Table) for 24h. Then, the wild type and mutant p3XFLAG-CSB-PGBD3 and p3XFLAG (as control) plasmids were transiently transfected into the HeLa cells using X-tremeGENE HP DNA Transfection Reagent (Roche). After culture for 24h, cells were exposed to UV irradiation (10J/m^2^). The cells were next harvested and counted, and reseeded into a new 6-well plate by 500 cells per well (three wells for duplicate) and incubated for 12 days. Media were then removed, and colonies stained with crystal violet. Cells were washed twice with PBS and the blue colonies were counted. This experiment was replicated three times. Data were expressed as percent survival relative to the control: [(average treated count)/(average control count)]x100%.

### H_2_O_2_ treatment and chromatin analysis

HeLa cells seeded in a 6 cm dish were transiently transfected with wild type and the 3 mutant CSB-PGBD3-eGFP plasmids using X-tremeGENE HP DNA Transfection Reagent (Roche) and cultured for 48 h. Then the cells were treated with H_2_O_2_ (5mM) at 4°C for 10 min in serum free medium and recovered at 0min, 15min, 30min, 1h, 2h, 3h, and 4h in complete medium at 37°C. Chromatin fractions were harvested as performed by Zou et al. [32] and analysed by western blot with antibody against GFP (Proteintech 66002-1-lg) and histone H3 (Merck Millipore 05–499).

### Immunoprecipitation

HEK293FT cells were transiently transfected with wild type and the 3 mutant p3XFLAG-CSB-PGBD3 plasmids using Lipo2000 (Invitrogen) and cultured for 24 h. Cells were then divided into three groups: without treatment; treatment with ultraviolet light (UV) irradiation (10 J/m^2^); treatment with H_2_O_2_ (5mM, 4°C, 10min). After treatment, protein of the cells was extracted using NETN buffer, and pulled down with anti-FLAG M2 magnetics beads (Sigma-Aldrich M8823). The interaction between CSB-PGBD3 and RNApol II was analyzed by western blot with the antibody against FLAG (Cell Signaling Technology 2368) and RNApol II (Santa Cruz Biotechnology sc-9001). All steps were performed at 4°C.

### Statistical analysis

The continuous data were checked for normality and described as mean ± SD or mean (95% confidence interval for mean). We calculated differences of clinical features between sporadic POF patients and controls using independent sample T-tests. For the mRNA expression of DNA repair factors, we calculated using Kruskal-Wallis one-way ANOVA on ranks, as well as a multiple comparison procedure using Dunnett’s method. For the percentage of cells in which RFP tagged protein was localized at the damaged sites, we calculated using Pearson chi-square test. We used SPSS (version 19) for all statistical analyses.

## Supporting Information

S1 Fig Plasmid construction of CSB-PGBD3-eGFP, CSB-PGBD3-RFP, Flag-CSB-PGBD3 and OGG1-GFP. The diagram of wild type and 3 mutant (ENST00000515869: c.643G>T, c.2237G>A, and c.3166G>A) plasmid of CSB-PGBD3-pEGFP-N1 (A), CSB-PGBD3-RFP (B), p3XFLAG-CSB-PGBD3 (C) and OGG1-pEGFP-N1 (D). Coding sequence of wild type CSB-PGBD3 was amplified by PCR (forward primer: 5’- CCCAAGCTTGCCACCATGCCAAATGA GGGAATCCCCCACT-3’; reverse primer: 5’- TGCTCTAGACTATTCAGTGTGATATTCAA-3’) with the vector pFLAG-HA-CSB-PGBD3-IREShyg3 as template, which was a kind gift from Alan M. Weiner (University of Washington, Seattle, WA). The amplicons were ligated between Hind III and Xba I sites of the expression vector pcDNA3.1 (Invitrogen). The mutations (c.643G>T, c.2237 G>A and c.3166G>A) were introduced by site-directed mutagenesis using QuikChange Lightning Site-Directed Mutagenesis Kit (Agilent Technologies) according to manufacturer’s instructions with the primers listed in S2 Table. To make CSB-PGBD3-eGFP and CSB-PGBD3-RFP expression vector, the coding sequences of wild type and mutant CSB-PGBD3 were amplified with respective pcDNA3.1 plasmid as template and primers listed in S3 Table and S4 Table, and the amplicons were ligated between Hind III and Xho I sites of the vector pEGFP-N1 (Clontech) and pSAT6-RFP-N1 (Clontech). To make the FLAG-CSB-PGBD3 expression vector, the coding sequences of wild type and mutant CSB-PGBD3 in vector pcDNA3.1 were cut and ligated into the vector p3XFLAG (Sigma) between Hind III and Xba I sites. Full length cDNA of human 8-oxoguanine DNA glycosylase type 1a (hOGG1-type 1a, nuclear form) was obtained by RT-PCR (forward primer: 5’- GAAGATCTATG CCTGCCCGCGCGCTTCTG-3’; reverse primer: 5’- GGCGACCGGTCTGCCTTCCGGCCCTTTGG AACC-3’), and OGG1-GFP expressing plasmid was then constructed by inserting hOGG1-type 1a cDNA into the pEGFP-N1 vector between Bgl II and Age I sites. All the constructs were confirmed by Sanger sequencing.
(TIF)

S1 Table Mutations of CSB-PGBD3 identified in 432 sporadic POF patients. (DOCX)

S2 Table CSB-PGBD3 mutagenesis primers. (DOCX)

S3 Table Primers used for amplification of wild type and mutant CSB-PGBD3 cloned into pEGFP-N1. (DOCX)

S4 Table Primers used for amplification of wild type and mutant CSB-PGBD3 cloned into pSAT6-RFP-N1. (DOCX)

S5 Table SiRNA used to silence CSB-PGBD3. The three siRNA for CSB-PGBD3 were mixed used to silence the gene in our study.
(DOCX)
