# Supplementary figures and images for: CSB-PGBD3 Mutations Cause Premature Ovarian Failure
Source: PLoS Genet. 2015 Jul 28;11(7):e1005419. doi: 10.1371/journal.pgen.1005419 (PMC4517778; doi:10.1371/journal.pgen.1005419)

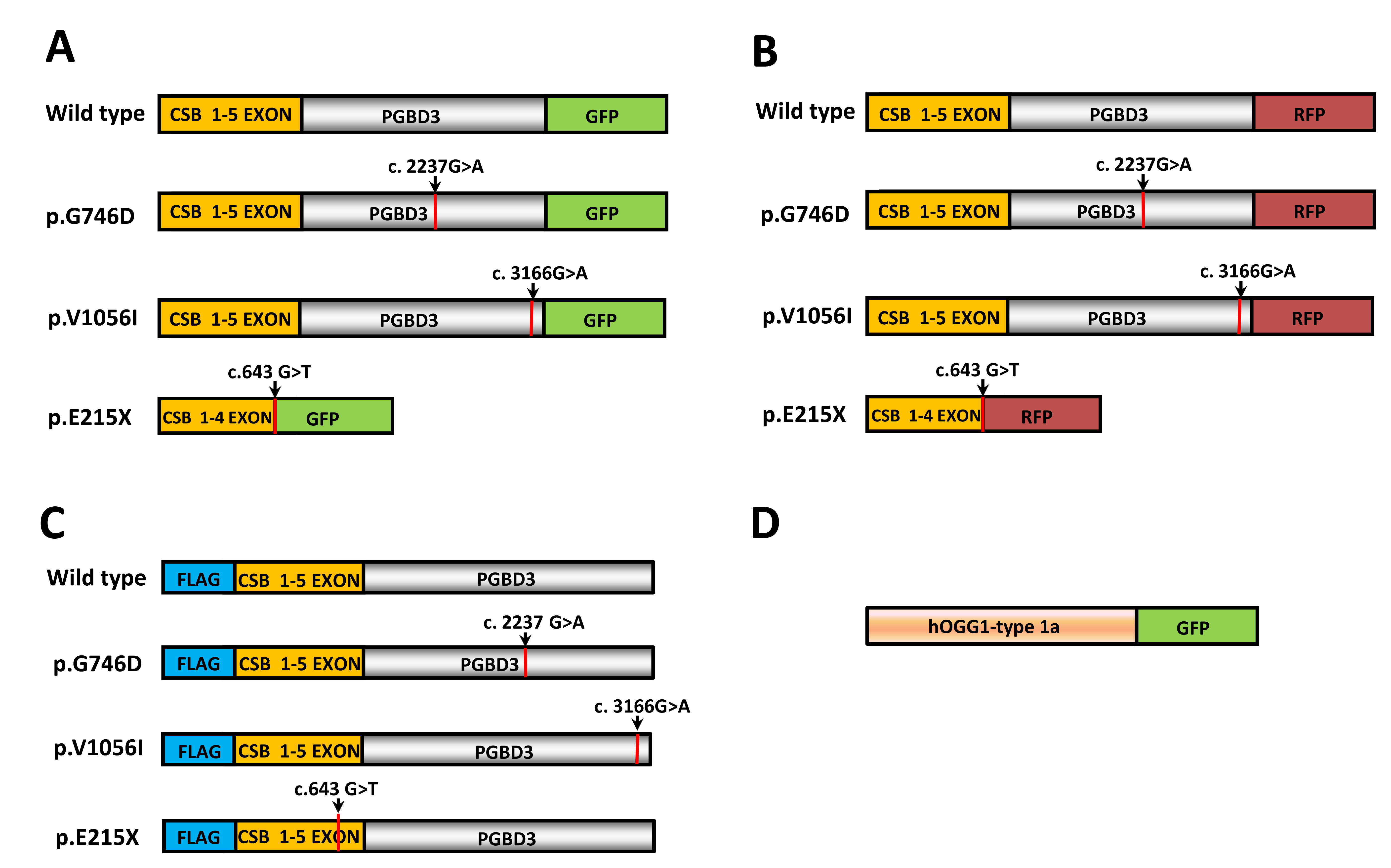

Supplement: S1 Fig — The diagram of wild type and 3 mutant (ENST00000515869: c.643G>T, c.2237G>A, and c.3166G>A) plasmid of CSB-PGBD3-pEGFP-N1 (A), CSB-PGBD3-RFP (B), p3XFLAG-CSB-PGBD3 (C) and OGG1-pEGFP-N1 (D). Coding sequence of wild type CSB-PGBD3 was amplified by PCR (forward primer: 5’- CCCAAGCTTGCCACCATGCCAAATGA GGGAATCCCCCACT-3’; reverse primer: 5’- TGCTCTAGACTATTCAGTGTGATATTCAA-3’) with the vector pFLAG-HA-CSB-PGBD3-IREShyg3 as template, which was a kind gift from Alan M. Weiner (University of Washington, Seattle, WA). The amplicons were ligated between Hind III and Xba I sites of the expression vector pcDNA3.1 (Invitrogen). The mutations (c.643G>T, c.2237 G>A and c.3166G>A) were introduced by site-directed mutagenesis using QuikChange Lightning Site-Directed Mutagenesis Kit (Agilent Technologies) according to manufacturer’s instructions with the primers listed in S2 Table. To make CSB-PGBD3-eGFP and CSB-PGBD3-RFP expression vector, the coding sequences of wild type and mutant CSB-PGBD3 were amplified with respective pcDNA3.1 plasmid as template and primers listed in S3 Table and S4 Table, and the amplicons were ligated between Hind III and Xho I sites of the vector pEGFP-N1 (Clontech) and pSAT6-RFP-N1 (Clontech). To make the FLAG-CSB-PGBD3 expression vector, the coding sequences of wild type and mutant CSB-PGBD3 in vector pcDNA3.1 were cut and ligated into the vector p3XFLAG (Sigma) between Hind III and Xba I sites. Full length cDNA of human 8-oxoguanine DNA glycosylase type 1a (hOGG1-type 1a, nuclear form) was obtained by RT-PCR (forward primer: 5’- GAAGATCTATG CCTGCCCGCGCGCTTCTG-3’; reverse primer: 5’- GGCGACCGGTCTGCCTTCCGGCCCTTTGG AACC-3’), and OGG1-GFP expressing plasmid was then constructed by inserting hOGG1-type 1a cDNA into the pEGFP-N1 vector between Bgl II and Age I sites. All the constructs were confirmed by Sanger sequencing. (TIF) [file pgen.1005419.s001.tif]
