# Supplementary material for: CSB-PGBD3 Mutations Cause Premature Ovarian Failure
Source: PLoS Genet. 2015 Jul 28;11(7):e1005419. doi: 10.1371/journal.pgen.1005419 (PMC4517778; doi:10.1371/journal.pgen.1005419)
Supplement: S1 Table — (DOCX) [file pgen.1005419.s002.docx]

**S1 Table.** **Mutations of CSB-PGBD3 identified in 432 sporadic POF patients.**

|  |  | **Genotype frequency** | |  | **Allele frequency** | |
| --- | --- | --- | --- | --- | --- | --- |
|  | **Mutation ID**  **ENST00000515869** | **Sporadic POF** | **Control** |  | **Sporadic POF** | **Control** |
|  |  | **(n, %)** | **(%)** |  | **(n, %)** | **(n, %)** |
|  | novel | GG(431,99.77) | - |  | G(863, 99.88) | - |
| **CSB**  **(Exon4)** | c.643G>T | GT(1, 0.23) | - |  | T(1,0.12) | - |
|  | p.E215X | TT(0,0) | - |  |  | - |
|  | novel | GG(431,99.77) | - |  | G(863, 99.88) | - |
| **PGBD3**  **(Exon1)** | c.3166G>A | AG(1, 0.23) | - |  | A(1, 0.12) | - |
|  | p.V1056I | AA(0,0) | - |  |  | - |
