# Supplementary material for: CSB-PGBD3 Mutations Cause Premature Ovarian Failure
Source: PLoS Genet. 2015 Jul 28;11(7):e1005419. doi: 10.1371/journal.pgen.1005419 (PMC4517778; doi:10.1371/journal.pgen.1005419)
Supplement: S2 Table — (DOCX) [file pgen.1005419.s003.docx]

**S2 Table. CSB-PGBD3 mutagenesis primers.**

| **Mutation** | **Primer** | **Sequence** |
| --- | --- | --- |
| **c.2237 G>A** | Forward | 5'-TGAATTCATGGTTCCTTATTTTGATCGTCACGGGTGCAAA-3' |
|  | Reverse | 5'-TTTGCACCCGTGACGATCAAAATAAGGAACCATGAATTCA-3' |
| **c.3166G>A** | Forward | 5'-GCCTTACATGTGAAGTGTTCCATTGAATATCACACTGAA-3' |
|  | Reverse | 5'-TTCAGTGTGATATTCAATGGAACACTTCACATGTAAGGC-3' |
| **c.643G>T** | Forward | 5'-CGCCAGTCTGGAGTAGGATGCAGAGCC-3' |
|  | Reverse | 5'-GGCTCTGCATCCTACTCCAGACTGGCG-3' |

Note: underline indicates the site of mutagenesis.
