# Supplementary material for: CSB-PGBD3 Mutations Cause Premature Ovarian Failure
Source: PLoS Genet. 2015 Jul 28;11(7):e1005419. doi: 10.1371/journal.pgen.1005419 (PMC4517778; doi:10.1371/journal.pgen.1005419)
Supplement: S3 Table — (DOCX) [file pgen.1005419.s004.docx]

**S3 Table. Primers used for amplification of wild type and mutant CSB-PGBD3 cloned into pEGFP-N1.**

| **Template** | **Primer** | **Sequence** |
| --- | --- | --- |
| **CSB-PGBD3-pcDNA3.1**  **Wild type** | Forward | 5'-CCGCTCGAGGCCACCATGCCAAATGAGGGAATC-3' |
|  | Reverse | 5'-CCCAAGCTTTTCAGTGTGATATTCAA-3' |
| **CSB-PGBD3-pcDNA3.1**  **with c.2237 G>A** | Forward | 5'-CCGCTCGAGGCCACCATGCCAAATGAGGGAATC-3' |
|  | Reverse | 5'-CCCAAGCTTTTCAGTGTGATATTCAA-3' |
| **CSB-PGBD3-pcDNA3.1**  **with c.3166G>A** | Forward | 5'-CCGCTCGAGGCCACCATGCCAAATGAGGGAATC-3' |
|  | Reverse | 5'-CCCAAGCTTTTCAGTGTGATATTCAA-3' |
| **CSB-PGBD3-pcDNA3.1**  **with c.643G>T** | Forward | 5'-CCGCTCGAGGCCACCATGCCAAATGAGGGAATC-3' |
|  | Reverse | 5'-CCCAAGCTTCTCCAGACTGGCGTGATC-3' |
