# Supplementary material for: CSB-PGBD3 Mutations Cause Premature Ovarian Failure
Source: PLoS Genet. 2015 Jul 28;11(7):e1005419. doi: 10.1371/journal.pgen.1005419 (PMC4517778; doi:10.1371/journal.pgen.1005419)
Supplement: S4 Table — (DOCX) [file pgen.1005419.s005.docx]

**S4 Table. Primers used for amplification of wild type and mutant CSB-PGBD3 cloned into pSAT6-RFP-N1.**

| **Template** | **Primer** | **Sequence** |
| --- | --- | --- |
| **CSB-PGBD3-pcDNA3.1**  **Wild type** | Forward | 5'-CCGCTCGAGGCCACCATGCCAAATGAGGGAATC -3' |
|  | Reverse | 5'- CCCAAGCTTGTTCAGTGTGATATTCAA -3' |
| **CSB-PGBD3-pcDNA3.1**  **with c.2237 G>A** | Forward | 5'- CCGCTCGAGGCCACCATGCCAAATGAGGGAATC -3' |
|  | Reverse | 5'- CCCAAGCTTGTTCAGTGTGATATTCAA -3' |
| **CSB-PGBD3-pcDNA3.1**  **with c.3166G>A** | Forward | 5'- CCGCTCGAGGCCACCATGCCAAATGAGGGAATC -3' |
|  | Reverse | 5'- CCCAAGCTTGTTCAGTGTGATATTCAA -3' |
| **CSB-PGBD3-pcDNA3.1**  **with c.643G>T** | Forward | 5'- CCGCTCGAGGCCACCATGCCAAATGAGGGAATC -3' |
|  | Reverse | 5'- CCCAAGCTTGCTCCAGACTGGCGTGATC -3' |
