# Supplementary material for: CSB-PGBD3 Mutations Cause Premature Ovarian Failure
Source: PLoS Genet. 2015 Jul 28;11(7):e1005419. doi: 10.1371/journal.pgen.1005419 (PMC4517778; doi:10.1371/journal.pgen.1005419)
Supplement: S5 Table — The three siRNA for CSB-PGBD3 were mixed used to silence the gene in our study. (DOCX) [file pgen.1005419.s006.docx]

**S5 Table. SiRNA used to silence CSB-PGBD3. The three siRNA for CSB-PGBD3 were mixed used to silence the gene in our study.**

| **siRNA name** |  | **Sequence** |
| --- | --- | --- |
| **Negative control** | sense | 5'- UUCUCCGAACGUGUCACGUTT -3' |
|  | antisense | 5'- ACGUGACACGUUCGGAGAATT-3' |
| **CSB-PGBD3-homo-512** | sense | 5'- GGUGGACAAUGCCAUCCAUTT -3' |
|  | antisense | 5'- AUGAUGGCAUUGUCCACCTT -3' |
| **CSB-PGBD3-homo-2711** | sense | 5'- GGAUCACAUUGACAGAGUUTT -3' |
|  | antisense | 5'- AACUCUGUCAAUGUGAUCCTT-3' |
| **CSB-PGBD3-homo-1636** | sense | 5'- GCAUAGAAGCAAGUGCUAUTT -3' |
|  | antisense | 5'- AUAGCACUUGCUUCUAUGCTT-3' |
